# Tumor-Associated Macrophages Promote Metastasis of Oral Squamous Cell Carcinoma via CCL13 Regulated by Stress Granule

**DOI:** 10.3390/cancers14205081

**Published:** 2022-10-17

**Authors:** Zhixin Liu, Tao Rui, Zhaoyu Lin, Shule Xie, Bin Zhou, Min Fu, Lianxi Mai, Chuandong Zhu, Guotao Wu, Youyuan Wang

**Affiliations:** 1 Department of Oral and Maxillofacial Surgery, Sun Yat-sen Memorial Hospital, Sun Yat-sen University, 107 Yanjiang West Road, Guangzhou 510120, China; 2 The Guangdong Provincial Key Laboratory of Malignant Tumor Epigenetics and Gene Regulation, Sun Yat-sen Memorial Hospital, Sun Yat-sen University, 107 Yanjiang West Road, Guangzhou 510120, China; 3 The Oral and Maxillofacial-Head and Neck Digital Precision Reconstruction Technology Research Center of Guangdong Province, Sun Yat-sen Memorial Hospital, Sun Yat-sen University, 107 Yanjiang West Road, Guangzhou 510120, China; 4 Department of Oral and Maxillofacial Surgery, Affiliate Stomatology Hospital of Guangzhou Medical University, Guangzhou Medical University, 31 Huangsha Avenue, Guangzhou 510000, China

**Keywords:** tumor-associated macrophage, OSCC metastasis, stress granule, CCL13, mRNA stability

## Abstract

**Simple Summary:**

Oral cancer is a devastating disease for humans. Tumor-associated macrophages (TAMs) are notorious tumor promoters. The major TAMs populations in most solid tumors are cancer-promoting M2 TAMs, which play a vital role in mediating the immunosuppressive tumor microenvironment, promoting tumor blood vessel formation and leading to tumor metastasis through the production of tumor-promoting inflammatory factors or chemokines. CCL13 is a crucial pro-tumor chemokine from M2 TAMs. G3BP1 has been reported to trigger tumor metastasis via mediating the cellular stress responses. Hitherto, it is unclear that how M2 TAMs released the CCL13 to promote OSCC metastasis. In the current study, we identified that G3BP1 can facilitate adaptive reactions of macrophages in response to adverse conditions in the tumor microenvironment, and these effects of G3BP1 simultaneously stimulated the expression of CCL13 from M2 TAMs, consequently improving oral cancer metastasis.

**Abstract:**

M2 tumor-associated macrophages (TAMs) have been a well-established promoter of oral squamous cell carcinoma (OSCC) progression. However, the mechanisms of M2 TAMs promoting OSCC metastasis have not been elucidated clearly. This study illustrated the regulatory mechanisms in which M2 TAMs enhance OSCC malignancy in a novel point of view. In this study, mass spectrometry was utilized to analyze the proteins expression profile of M2 type monocyte-derived macrophages (MDMs-M2), whose results revealed the high expression of G3BP1 in M2 macrophages. RNA sequencing analyzed the genome-wide changes upon G3BP1 knockdown in MDMs-M2 and identified that CCL13 was the most significantly downregulated inflammatory cytokines in MDMs-M2. Co-immunoprecipitation and qualitative mass spectrometry were used to identify the proteins that directly interacted with endogenous G3BP1 in MDMs-M2. Elevated stress granule (SG) formation in stressed M2 TAMs enhanced the expression of CCL13, which promoted OSCC metastasis both in vitro and in vivo. For mechanisms, we demonstrated SG formation improved DDX3Y/hnRNPF-mediated CCL13 mRNA stability, thus enhancing CCL13 expression and promoting OSCC metastasis. Collectively, our findings demonstrated for the first time the roles of CCL13 in improving OSCC metastasis and illustrated the molecular mechanisms of CCL13 expression regulated by SG, indicating that the SG-CCL13 axis can be the potential targets for TAM-navigated tumor therapy.

## 1. Introduction

Oral cancer is one of the most lethal tumors worldwide [1]. OSCC is the primary histopathological type of oral cancer, and thousands of OSCC patients die from regional or distant metastasis of oral cancer annually [2,3]. We previously reported that the cytokine TNF-α from the tumor microenvironment (TME) promoted tongue squamous cell carcinoma (TSCC) metastasis by activating cancer-associated fibroblasts (CAFs) and enhancing their release of SDF-1 [4]. Macrophages were the primary cell populations secreting TNF-α [5], and macrophage-mediated inflammatory responses have been demonstrated to facilitate cancer metastasis [6].

TAMs populations had also been famous for their diversity in phenotypes and functions [7]. Macrophages cultured with lipopolysaccharide (LPS) and IFN-γ or with IL-4 and IL-13 will polarize to M1 or M2 macrophages, respectively [8]. Recently, the advances in single-cell sequencing technique have enhanced our recognition of TAMs subtypes [9]. However, only spatial analysis supported by multiplexed immunohistochemistry (mIHC) techniques can exactly show the TAMs spatial distributions in situ without impairing their actual distributions in tumor tissues [10]. TAMs were responsible for many malignant behaviors of cancer. On the one hand, as one of immunosuppressive myeloid cells, TAMs could promote tumor immune escape through inducing an immunosuppressive tumor microenvironment [11,12]. They not only inhibited the adaptive antitumour immune responses, but also blocked the innate immune system activities to facilitate tumor immune escape. PD-L1 in tumor cells or TAMs is an important mechanism for tumor immune escape via suppressing the activation of cytotoxic T lymphocytes [13,14,15]. TAMs have also been blamed for tumor escape from the phagocytosis, as well as the detection and clearance of malignant cells mediated by macrophages via the innate immune checkpoints expressing in TAMs (PD-1, SIRPα, Siglec-10, etc.) [16,17,18]. On the other hand, M2 TAMs were also well-established promoters of tumor progression [6,19,20]. The triple molecular chaperones of CDC37/HSP90α/β from oral cancer-derived extracellular vesicles (EVs) were found to facilitate tumor progression via inducing epithelial-mesenchymal transition (EMT) and M2 TAMs polarization [21]. TAMs mediated cancer cell stemness of head and neck squamous cell carcinoma (HNSCC) by increasing availability of hyaluronic acid (HA) and influencing PI3K-4EBP1-SOX2 pathway [22]. M2 TAMs have been identified to be precursors of OSCC progression and both CD163^+^ TAMs and CD206^+^ TAMs were correlated with clinical poor prognosis of OSCC [23,24,25]. However, the mechanisms in which M2 TAMs improved OSCC metastasis have not been thoroughly illustrated.

TME was an adverse environment for cells survival as result of its environmental stressors, such as oxidative stress, hypoxia, or nutrient deprivation [26]. The global translation level in cells under stresses was limited to preserve energy for cells survival [27]. One of well-established stress-induced responses was stress granule (SG) formation which consists of ribonucleoprotein complex via sequestrating translationally stalled mRNA, stress-associated RNA-binding proteins and eukaryotic translation initiation factors [28,29]. SG was a form of membraneless organelles in the cytoplasm [30], and it was also a vital structure for mRNA storage and stability, as well as reprogramed mRNA translation during cell stress periods. SG can improve the stability of transcripts stored in ribonucleoprotein complex and allow rapid restoration of protein synthesis after the termination of cell stress responses [31]. However, some mRNAs encoding chaperones or repair enzymes were usually excluded from SG for their continual translation to sustain crucial bioprocesses in response to cell stress [27,32]. In many studies on the relationship between SG and diseases, SG was associated with tumor initiation and progression [33,34].

In the current study, we identified the infiltration of CD206^+^TAMs were correlated with tumor metastasis and poor prognosis of OSCC patients. SG formation regulated the expression of CCL13 in M2 TAMs through enhancing DDX3Y/hnRNPF-mediated CCL13 mRNA stability to promote OSCC metastasis. These findings may provide a novel understanding of the mechanisms by which M2 TAMs released protumor chemokines to facilitate OSCC metastasis.

## 2. Materials and Methods

Additional information was provided in the Supplementary Materials and Methods.

### 2.1. Cell Culture

The culture methods of human OSCC cell lines (CAL27 and SAS cells) and macrophages were described in the Supplementary Materials and Methods.

### 2.2. Patients and Specimens

A total of 48 patients diagnosed as OSCC with or without lymph node metastasis in our center between 2016 and 2021 were recruited for our research. All tissue specimens and adjacent noncancerous specimens were obtained via tumor resection surgery. This study has been approved by the research ethics committee of Sun Yat-sen Memorial hospital, Sun Yat-sen University (Approval numbers: SYSKY-2022-322-01). All patients who provided specimens gave informed consent. Clinical data were obtained by chart review and confirmed by experienced surgeons. The mean follow-up time of all patients were 27.1 months.

### 2.3. Multiplex Immunohistochemistry (mIHC)

We performed mIHC of human OSCC tissues using an Opal 7-color manual IHC kit (NEL801001KT, PerkinElmer, MA, USA) according to the manufacturer’s guides and the methods from another study [12]. Data were analyzed via the software Vetra Polaris (PerkinElmer).

### 2.4. Immunohistochemistry (IHC) and Immunofluorescence Staining (IF) of Tumor Tissues

The human or mouse primary tumor specimens were used for immunohistochemistry and immunofluorescence analysis. The analysis of immunohistochemical results with Image J included two steps. On the one hand, the IHC Profiler plugin was used to automatically score the staining of the samples. IHC Profiler, which simultaneously took the average gray value (staining intensity) and the percentage of positive area (staining area) of positive cells as IHC measurement indicators, finally gave us four scores: High positive (3+), Positive (2+), Low Positive (1+) and Negative (0). On the other hand, using the Trainable Weka Segmentation plugin, the positive and negative cells were counted separately based on machine learning algorithms. Other experimental details can be seen as described in the Supplementary Materials and Methods.

### 2.5. Cell Immunofluorescence (IF)

The cell IF staining of macrophages were performed as shown in the Supplementary Materials and Methods.

### 2.6. Flow Cytometry

The monocyte-derived macrophages were detected via flow cytometry as shown in the Supplementary Materials and Methods.

### 2.7. siRNA Transfection for Macrophages

The specific siRNAs were transfected into macrophages as revealed in the Supplementary Materials and Methods.

### 2.8. Plasmid Construct

The methods to generate G3BP1-knockdown plasmid constructs have been described in the Supplementary Materials and Methods.

### 2.9. Transwell Migration Assay and Wound Healing Assay

The co-culture model between M2 macrophages and CAL27/SAS cells using transwell devices were consisted of 0.8μm-chambers (upper chambers) and the 24-well cell culture plates (lower chambers). The M2 macrophages were inoculated and cultured in lower chambers 24 h before the co-culture experiment. On the onset of formal experiment, the CAL27/SAS cells were inoculated in the upper chambers. Then, the upper chambers covered with CAL27/SAS cells would be placed into the lower chambers with macrophages (avoiding the formation of air bubble at the interface between upper and lower chambers) and continued to culture 24 h. Further details of the experiment can be seen in the Supplementary Materials and Methods.

### 2.10. ELISA

These experiment details can be seen in the Supplementary Materials and Methods.

### 2.11. Real-Time Quantitative PCR (RT-qPCR) and Western Blot

Total RNA, cDNA synthesis and qPCR were performed as previously described [35]. The primers sequences were shown as Supplementary Table S2. Western blot analysis of cells can be seen in the previous study [35]. All the whole western blot figures can be found in the supplementary materials.

### 2.12. RNA Sequencing

Details about RNA sequencing have been listed in the Supplementary Materials and Methods.

### 2.13. Coimmunoprecipitation and Mass Spectrometry

The methods to perform coimmunoprecipitation and mass spectrometry have been shown in the Supplementary Materials and Methods.

### 2.14. Fluorescence In Situ Hybridization (FISH)

FISH were performed in macrophages followed by immunostaining of SG nucleating protein. Methods concerned can be seen in the Supplementary Materials and Methods.

### 2.15. RNA Immunoprecipitation (RIP)

RIP experiments were performed using the Magna RIP RNA-Binding Protein Immunoprecipitation Kit (Millipore, Billerica, MA, USA) according to the manufacturer’s instructions. Antibody against hnRNPF, as well as normal Rabbit IgG (3900S, as isotype control) were used for IP. The coprecipitated RNAs were detected by RT-qPCR. The total RNAs were used as the input controls.

### 2.16. RNA Stability Assay

RNA stability assays were performed as previously described [36]. Cells were incubated with 5 µg/mL actinomycin D (Sigma Aldrich, A1410, Shanghai, China) over the indicated time courses prior to RNA extraction and RT-qPCR analysis.

### 2.17. H&E Staining

H&E staining was performed using Hematoxylin-Eosin staining Kit (Meilunbio, MB9898) according to manufacturer’s instruction. The area of the xenograft lesions in mice tongue was calculated using image J software. The workflow for area measurement of metastatic tumors in mice neck requires two steps: 1. Select the region of interest (ROI) and perform image segmentation; 2. Measure the area of positive staining parts. Use tools such as rectangle, circle or other ways to select the parts of images wanted by us, and then Measure their areas according to the tips of software. A formula was used to compare the percent of xenografts area to the area of whole tongue tissues in different group: (areas of xenografts/areas of tongue) × 100%.

### 2.18. Orthotopic Metastasis Model in the Mouse Tongue

The BALB/c nude mice were used to build up orthotopic xenografts model in tongue according to the previous study [20]. These methods have been described in the Supplementary Materials and Methods.

### 2.19. Statistical Analysis

All statistical analyses were conducted using SPSS 26.0 software (SPSS Inc., Chicago, IL, USA). Major experimental graphs were produced with GraphPad Prism 8.0. One-way ANOVA was used to compare G3BP1, CCL13 and CD206 expression in OSCC specimens. The Kruskal–Wallis test and χ2 test were used to examine the relationships between clinicopathological characteristics and CCL13 protein expression. Survival curves were plotted using the Kaplan–Meier method and compared with the log-rank test. The one-way or two-way ANOVAs were used to compare the results from RT-qPCR, transwell migration assays, wound healing assays, the tumor xenograft models and other experiments between the different groups. Unless otherwise noted, quantitative data are expressed as the mean and standard error of the mean (S.E.M.) values. It was considered statistically significant when the *p* < 0.05 (*, *p* < 0.05; **, *p* < 0.01 and ***, *p* < 0.001) compared with the control.

## 3. Results

### 3.1. M2 TAMs Correlated with Metastasis and Poor Prognosis of OSCC Patients

**Figure 1:**
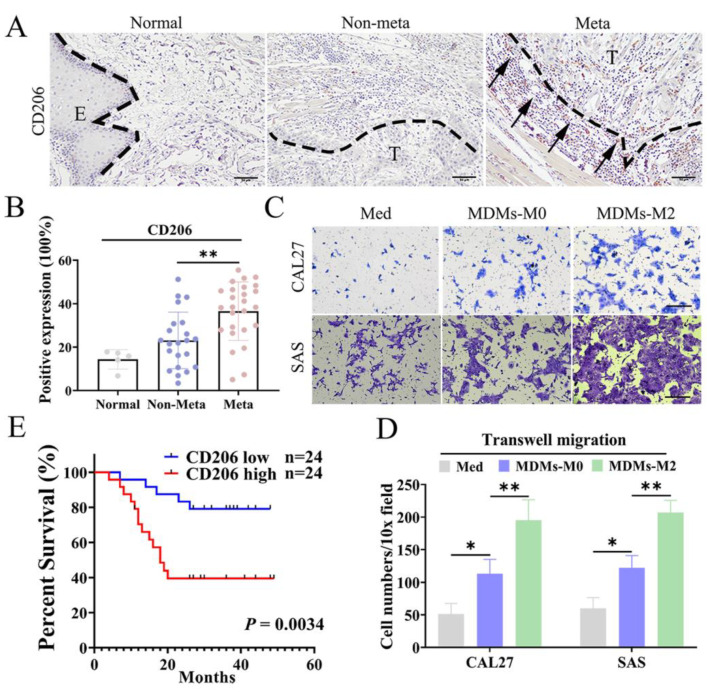
M2 TAMs enhanced OSCC metastasis and were correlated with poor prognosis of OSCC patients. (**A**) Representative images of CD206 staining in non-metastasis, metastasis, and paired para-tumor tissues from same patients. The positive staining has been highlighted by black arrows. E: Epithelium um, T: Tumor nest. Scale bar: 50 um. (**B**) Statistical results of CD206 positive expression in 26 OSCC specimens with neck lymph node metastasis and 22 without metastasis. (**C**,**D**) Transwell migration assays of CAL27 and SAS cells co-culturing with indicated macrophages were performed and statistical results were shown by the bar graph. Scale bar: 50 um. (**E**) Survival curves of 48 OSCC patients with low or high CD206 expression. The *p* value was determined by log-rank test. All experiments were performed in triple times, and the data were presented as the means ± SD. (*, *p* < 0.01 and **, *p* < 0.01).

CD206, a well-established TAM marker, was used to define M2 TAMs in this study. Immunohistochemical data showed that the expression of CD206 in metastatic OSCC tissues were significantly higher than those in OSCC tissues without lymph node metastasis (Figure 1A,B). Subsequently, we investigated the impacts of the M2 TAMs on OSCC cells in vitro. The monocyte-derived macrophages (MDMs) were induced using the CD14-positive monocytes from human peripheral blood. We validated the M2 type MDMs (MDMs-M2) by detecting relevant M2 TAMs markers (Figure S1). A co-culture model was established to investigate the reciprocal interaction between macrophages and OSCC cells. CAL27 and SAS cells co-culturing with MDMs-M2 exhibited greater mobility compared with the control group (Figure 1C,D). The Kaplan–Meier analysis revealed high expression of CD206 in OSCC tissues was correlated with lower overall survival rate of OSCC patients (Figure 1E). It demonstrated the roles of CD206 in the prognosis of OSCC patients. According to these data, we concluded that M2 TAMs could promote OSCC metastasis and were correlated with poor prognosis of OSCC patients.

### 3.2. Inhibition of SG Formation Attenuated the Protumor Effects of M2 TAMs

**Figure 2:**
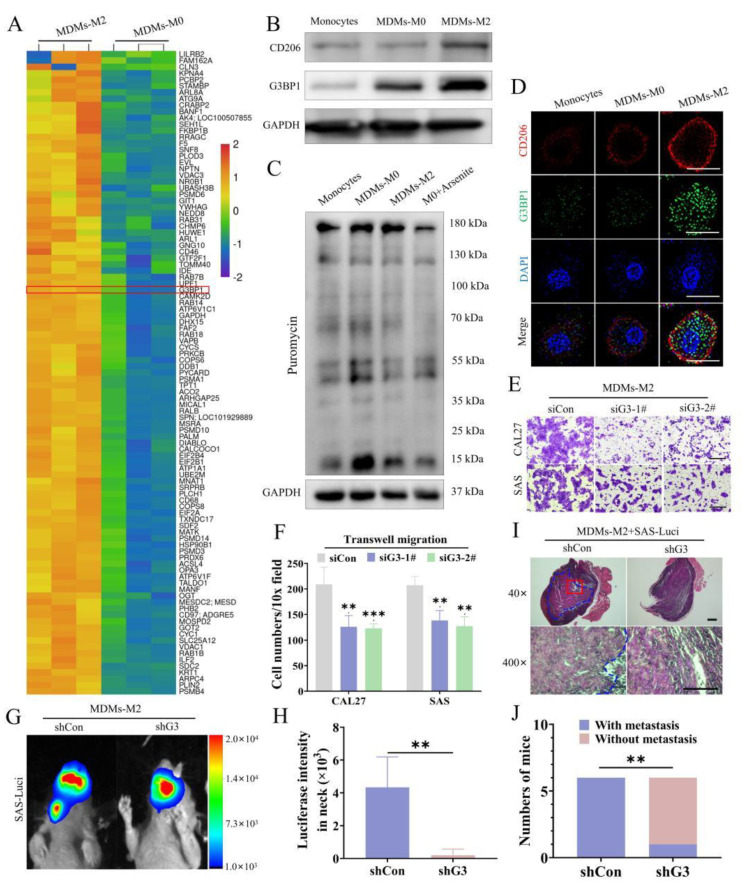
Increased SG formation in M2 TAMs promoted OSCC metastasis. (**A**) The data from heat map showed upregulated G3BP1 expression in MDMs-M2 compared with MDMs-M0 via Mass Spectrometry. (**B**) Western blot analysis demonstrated the upregulated protein expression of G3BP1 in MDMs-M2. (**C**) The indicated macrophages were incubated with puromycin (30 µg/mL) for 20 min before lysis, and followed by analysis of Western blot with a puromycin-specific antibody to detect the global translation levels. (**D**) IF images revealed increased SG in MDMs-M2. Scale bar, 10 μm. (**E**,**F**) Cell migration assays of CAL27 and SAS cells co-culturing with MDMs-M2 transfected with control or G3BP1 siRNA using Transwell inserts. Scale bar, 50 μm. (**G**,**H**) SAS cells stably expressing the luciferase and mixed with MDMs-M2 transfected with control or G3BP1 shRNA were injected into the tongue of BALB/c nude mice (*n* = 6). Bioluminescence signals of metastasis tumor (neck lymph node) were quantified on 18 days after tumor transplant with an in vivo imaging system. (**I**) H&E staining of the neck lymph nodes from mice. Blue dotted lines: metastasis tumor lesions. Scale bars, 100 μm. The enlarged pictures are placed on the bottom. Scale bars, 30 μm. (**J**) The numbers of mice with and without neck lymph nodes metastasis. All experiments were performed in triplicate, and the data were presented as the means ± SD. (**, *p* < 0.01; and ***, *p* < 0.001).

Although the pro-tumor effects of M2 TAMs have been verified, the reasons why the M2 TAMs can be a promoter of OSCC metastasis should be further investigated. We performed mass spectrometry with M2 TAMs and observed that a cluster of stress-associated proteins were upregulated in MDMs-M2. Many of the differentially expressed proteins (Figure 2A) were mainly involved in integrated stress response (ISR), especially SG assembly [37,38]. SG formation was triggered by SG nucleator proteins, such as G3BP1. SG can be visualized by detecting G3BP1 [39,40]. We demonstrated the high expression of G3BP1 in MDMs-M2 (Figure 2B). SG assembly was also deemed as a result of inhibited translation initiation [41]. We used ribopuromycylation to quantify global translation level [42] and observed that global translations were limited in MDMs-M2 (Figure 2C). Additionally, we demonstrated increased SG formation in MDMs-M2 (Figure 2D).

We wondered if SG formation played a pivotal role in sustaining the protumor phenotype of M2 TAMs. SG formation can be inhibited by depletion of G3BP1 (Figure S2A–C). Inhibition of SG formation downregulated CD206 expression in MDMs-M2 (Figure S2D,E). Cycloheximide (CHX) was used as a positive control to block SG assembly. Simultaneously, the effects of SG on OSCC metastasis were also testified. Compared with the control group, G3BP1 knockdown inhibited their pro-tumor effects of MDMs-M2 in vitro and in vivo (Figure 2E–J). These results highlighted the roles of SG formation in enhancing the pro-metastatic efficacy of M2 TAMs.

### 3.3. Disruption of SG Decreased the Production of CCL13 in M2 TAMs

**Figure 3:**
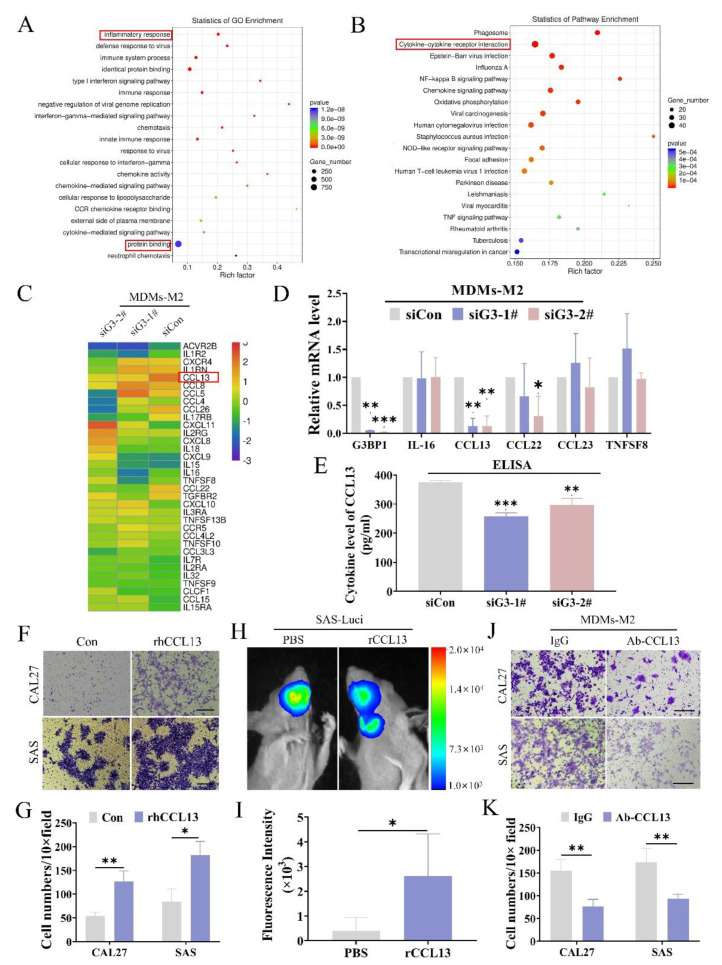
High-throughput sequencing analysis of differential expression of cytokines in G3BP1-depleted MDMs-M2. (**A**,**B**) Go Enrichment and KEGG analysis of differential genes upon after G3BP1-knockdown in MDMs-M2. (**C**) Heat map of differential cytokines genes data revealed the downregulated cytokines in G3BP1-depleted MDMs-M2. (**D**) RT-qPCR confirmed the upregulated CCL13 mRNA expression in MDMs-M2 transfected with control or G3BP1 siRNA. (**E**) The decreased production of CCL13 from MDMs-M2 after transfected by control siRNA or G3BP1 siRNA were testified by ELISA assays. (**F**,**G**) Transwell assays of CAL27 and SAS cells treated by 0 or 40 ng/mL recombinant human CCL13 cytokines. Statistical analysis results were shown as a bar graph. Scale bar, 50 μm. (**H**,**I**) SAS cells were injected into the tongue of BALB/c nude mice (*n* = 4) and followed by treatment of recombinant CCL13 cytokines. Bioluminescence signals of metastasis tumors (neck lymph nodes) were quantified with an in vivo imaging system. (**J**,**K**) Transwell migration assays of CAL27 and SAS cells coculturing with indicated macrophages were shown, and the neutralized antibody of CCL13 were added into Transwell inserts. Scale bar, 50 μm. All experiments were performed in triplicate, and the data were presented as the means ± SD. (* *p* < 0.01, **, *p* < 0.01, and ***, *p* < 0.001).

We have demonstrated that SG formation enhanced the pro-metastatic abilities of M2 TAMs above. However, the mechanisms by which SG formation regulated these biological processes remained unknown. Here, we used MDMs-M2 transfected with control and G3BP1 siRNAs to perform RNA sequencing. The Venn diagram revealed 901 differentially expressed genes in two siRNA groups (Figure S3). Most of the differentially expressed genes participated in the biological processes “inflammatory response” and “cytokine-cytokine receptor interaction” according to the Gene Ontology (GO) and Kyoto Encyclopedia of Genes and Genomes (KEGG) analysis (Figure 3A,B). We suspected SG formation may regulate the production of cytokines in MDMs-M2. Among the differentially expressed cytokines, CCL13 was the dominantly highly expressed and most significantly downregulated in G3BP1-knockdown groups (Figure 3C). We also testified the downregulation of CCL13 after G3BP1 depletion in MDMs-M2 (Figure 3D,E). We treated OSCC cells with recombinant human (rh) CCL13 proteins (rhCCL13), which enhanced OSCC metastasis in vitro and in vivo (Figure 3F–I). We then adopted monoclonal anti-CCL13 antibodies to neutralize the effects of CCL13 and the OSCC cells mobility was markedly inhibited (Figure 3J,K). These findings suggested that SG regulated the production of CCL13 in MDMs-M2 to promote OSCC metastasis.

### 3.4. The SG Mediated the Direct Connection between G3BP1 and DDX3Y-hnRNPF to Regulate the Expression of CCL13

**Figure 4:**
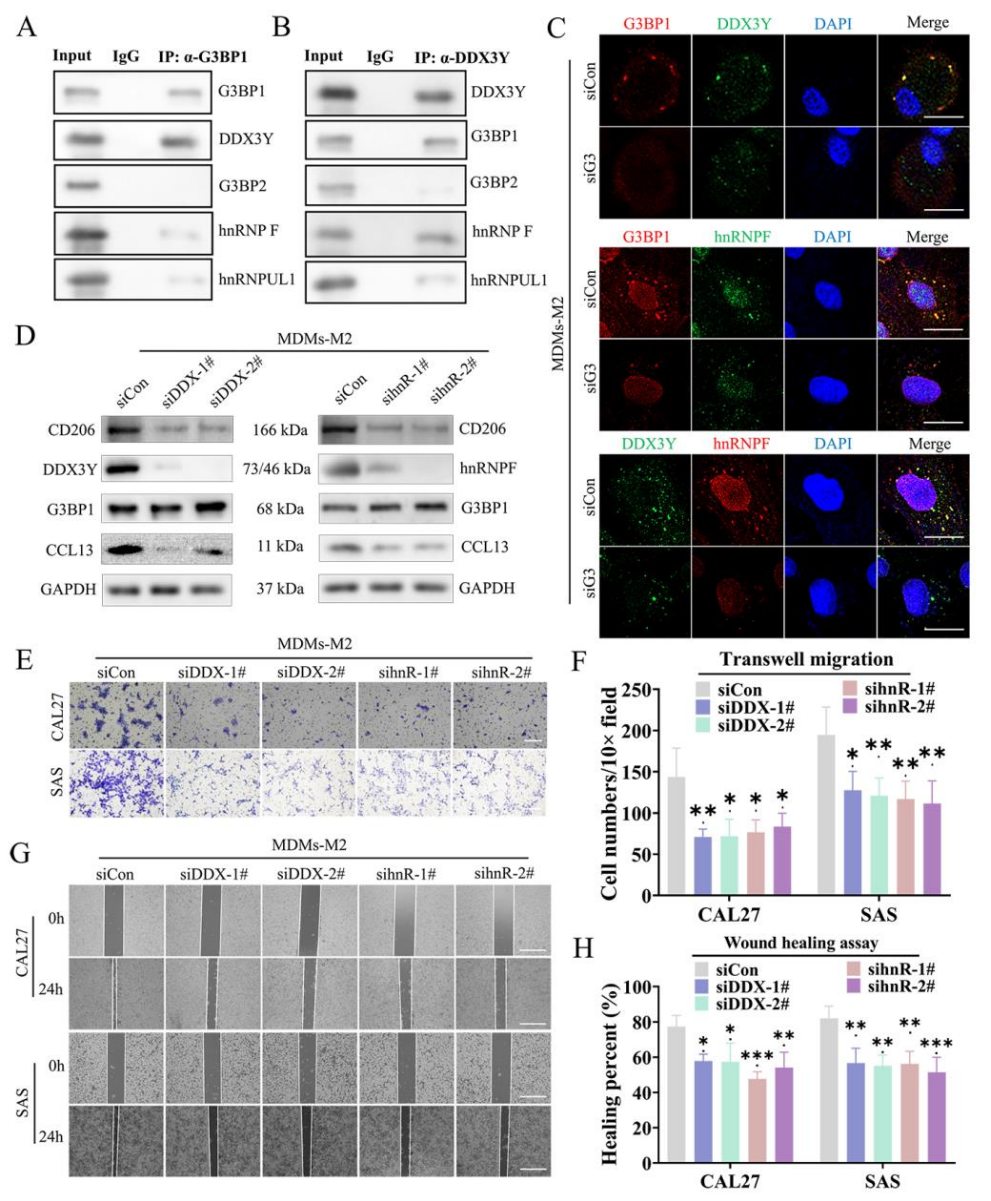
SG regulated the interaction between G3BP1 and DDX3Y-hnRNPF and the expression of CCL13. (**A**,**B**) The lysates from MDMs-M2 were performed to coimmunoprecipitation using rabbit isotype IgG or G3BP1 and DDX3Y antibody. (**C**) The representative immunostaining images of G3BP1 and DDX3Y or hnRNPF were performed by IF in MDMs-M2 transfected with control or G3BP1 siRNA. Scale bar, 10 μm. (**D**) Immunoblotting analyzed the protein expression of CCL13 and CD206 in MDMs-M2 transfected with control or DDX3Y and hnRNPF siRNA. (**E**,**F**) Transwell assays of CAL27 and SAS cells co-cultured with MDMs-M2 transfected with control siRNA or DDX3Y and hnRNPF siRNA. Scale bar, 50 μm. (**G**,**H**) Wound healing assays of CAL27 and SAS cells treated by conditioned medium from MDMs-M2 transfected with control siRNA or DDX3Y and hnRNPF siRNA. Scale bar, 100 μm. All data were derived from three independent experiments. (*, *p* < 0.05; **, *p* < 0.01 and ***, *p* < 0.001).

GO analysis showed that the differentially expressed genes upon G3BP1 knockdown in MDMs-M2 were involved in the biological processes of “protein binding” (Figure 3B). SG has been reported to mediate various cell bioprocesses through sequestering some important proteins or mRNAs into the shields or cores of SG [38]. We wondered whether the SG dynamics was linked to CCL13 expression. Here, we performed coimmunoprecipitation followed by mass spectrometry to identify proteins interacting with G3BP1 in MDMs-M2. A total of 138 candidate proteins were pulled down by endogenous G3BP1 in MDMs-M2 (Figure S4A,B). Several proteins related to “response to stimulus” were screened out for further study (Table S1). Subsequently, we proved that G3BP1 interacted directly with DDX3Y, whereas DDX3Y binded directly to hnRNPF, facilitating the formation of G3BP1-DDX3Y-hnRNPF complex (Figure 4A,B). We also observed that both DDX3Y and hnRNPF colocalize with G3BP1, whereas inhibition of SG formation disrupted their colocalization in SG (Figure 4C). These results implied the connection between G3BP1 and DDX3Y/hnRNPF was regulated by SG formation. To confirm whether the G3BP1-DDX3Y-hnRNPF complex mediated by SG regulated the expression of CCL13, we blocked the interaction between G3BP1 and DDX3Y/hnRNPF by depleting the expression of DDX3Y and hnRNPF. Knockdown of DDX3Y and hnRNPF impaired the expression of CD206 and CCL13 (Figure 4D). Moreover, the migration of CAL27 and SAS cells co-culturing with MDMs-M2 transfected with DDX3Y and hnRNPF siRNA was blocked compared with controlled group (Figure 4E–H). Collectively, SG mediated the interaction between G3BP1 and DDX3Y-hnRNPF to enhance the expression of CCL13.

### 3.5. SG Enhanced DDX3Y/hnRNPF-Mediated CCL13 mRNA Stability via Binding with Its Transcripts

**Figure 5:**
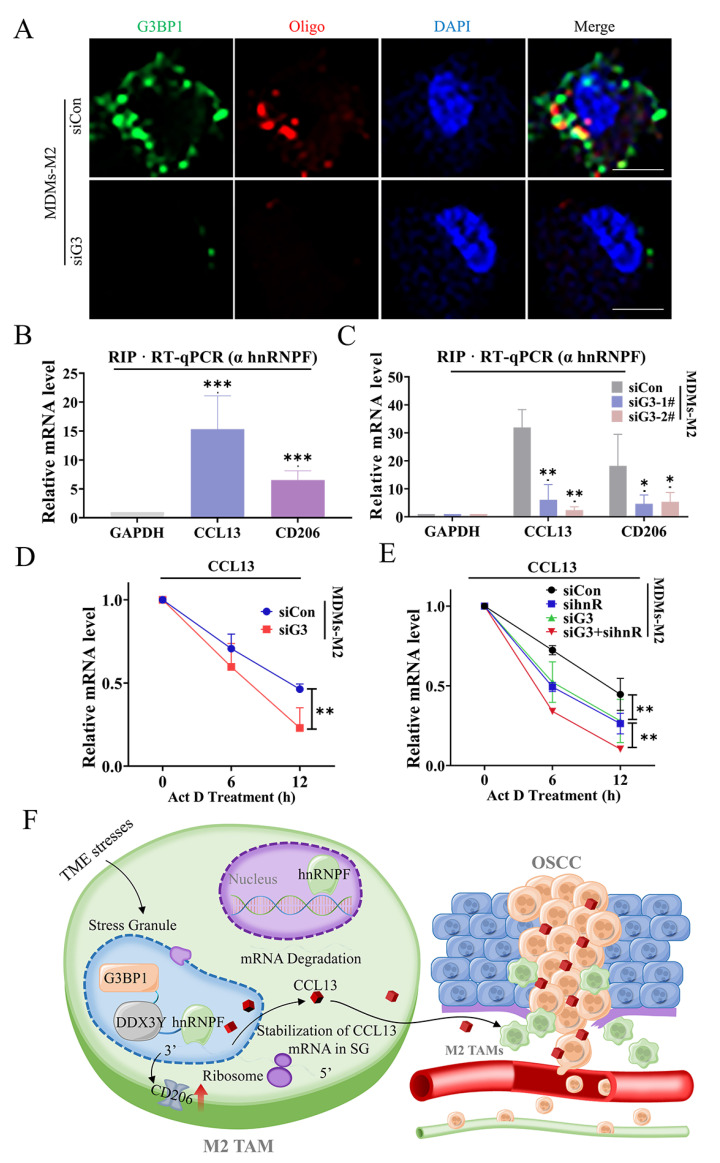
SG formation enhanced DDX3Y/hnRNPF-mediated mRNAs stability via binding with these transcripts. (**A**) Representative images of mRNA FISH detecting CCL13 mRNA and immunofluorescence of G3BP1. Scale bar, 10 μm. (**B**) RIP was performed with MDMs-M2 lysates using antibody against hnRNPF. Bar chart showed RT-qPCR data for the indicated transcripts. (**C**) RIP was performed using lysates from MDMs-M2 transfected with control or G3BP1 siRNA via antibody against hnRNPF. The bar chart showed results of the analysis of the indicated transcripts. (**D**) The degradation of CCL13 mRNA was determined by RT-qPCR after treatment with actinomycin D (5 μg/mL) for the indicated times in MDMs-M2 following G3BP1 knockdown. (**E**) The assays of CCL13 mRNA stability were performed by RT-qPCR for the indicated times in MDMs-M2 followed by knockdown of G3BP1 and hnRNPF. (**F**) The working model elucidated how M2 TAMs released CCL13 to promote OSCC metastasis. The SG formation enhanced the DDX3Y/hnRNPF-dependent mRNA stability of CCL13 and CD206. TME stressors facilitated SG formation, thus sequestering CCL13 and CD206 mRNA into SG via binding with hnRNPF. The mRNA stability of CCL13 and CD206 were improved in SG. which increased the expression of CD206 and CCL13 in TAMs. Finally, the CCL13 from M2 TAMs promoted OSCC metastasis. (*, *p* < 0.05; **, *p* < 0.01 and ***, *p* < 0.001).

Although SG formation was usually accompanied by limited global translation [43], SG-associated translation has been reported and whole translation cycles of transcripts localizing in SG can been accomplished [44]. These data of fluorescence in situ hybridization (FISH) demonstrated that some of CCL13 mRNA were localized in G3BP1^+^ SG (Figure 5A), implying some of CCL13 mRNA were sequestered into SG. However, the ways of CCL13 mRNA moving into SG were still not clear.

hnRNPF was an RNA binding protein correlating with tumor progression [45]. We needed to confirm whether the RNA binding activities of hnRNPF regulated the expression of CD206 and CCL13. Data from RNA immunoprecipitation (RIP) followed by RT-qPCR showed that hnRNPF interacted with rich mRNA of CCL13 and CD206 (Figure 5B). However, inhibition of SG formation decreased the abundance of CCL13 and CD206 binding with hnRNPF (Figure 5C), indicating that SG formation mediated the RNA binding activity of hnRNPF to regulate the expression of these transcripts. hnRNPF has been reported to regulate mRNA alternative splicing or the stability of specific mRNA transcripts by binding to the 3′ UTRs of genes [45,46]. The mRNA stability assay indicated that the stabilities of CCL13 and CD206 mRNA were higher in control MDMs-M2 than those in hnRNPF-depleted MDMs-M2 (Figure S5A,B). We treated G3BP1-knockdown MDMs-M2 with actinomycin D and discovered that G3BP1 depletion decreased the stability of CCL13 and CD206 mRNA, demonstrating SG formation had positive effects on hnRNPF-mediated stabilization of CD206 and CCL13 mRNA (Figure 5D). Disruption of G3BP1 expression further decreased the stability of CD206 and CCL13 mRNA in hnRNPF-knockdown MDMs-M2 (Figure 5E). Finally, we depicted a schematic diagram to clarify all works performed in this study (Figure 5F). These data supported the hypothesis that SG formation enhanced DDX3Y/hnRNPF-mediated stabilization of CD206 and CCL13 mRNA, thus elevating CCL13 expression and promoting OSCC metastasis.

### 3.6. SG Formation in M2 TAMs Promoted OSCC Metastasis In Vivo

**Figure 6:**
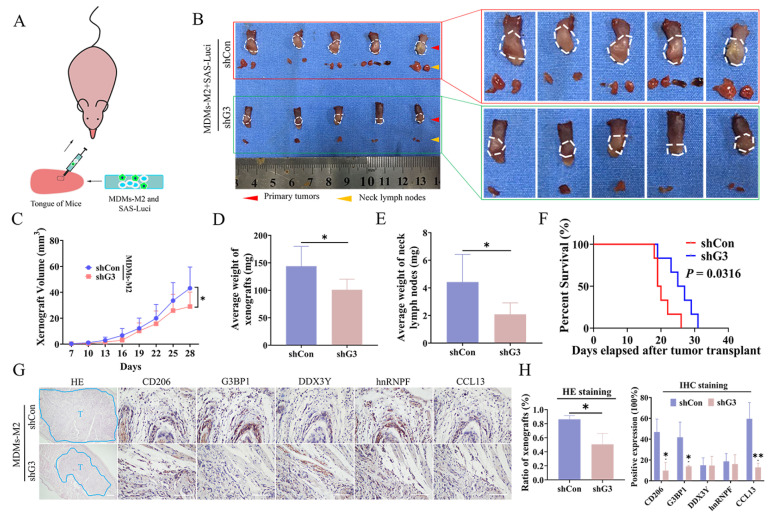
CCL13 regulated by SG formation in M2 TAMs promoted OSCC metastasis in vivo. (**A**) The schematic diagram of orthotopic metastasis model in BALB/c nude mice. SAS cells stably expressing the luciferase and mixed with MDMs-M2 transfected with control or G3BP1 shRNA were injected into the tongue of mice. (**B**) The sizes of primary tumor or neck lymph nodes on 28 days after tumor injection in control or G3BP1-depleted group. The enlarged pictures were also inserted at the right side. (**C**) Xenograft growth statistics on 28 days after tumor injection in control or G3BP1-depleted group. (**D**) The average weight of transplanted tumors in control or G3BP1-depleted group. (**E**) The mean weight of lymph nodes in control or G3BP1-depleted group. (**F**) Kaplan–Meier survival curves were used to assess the overall survival rate of mice (*n* = 6) with tumors, and a log-rank test was used to calculate the statistical significance. (**G**) Representative images of IHC staining showed lower expression of CCL13 and CD206 in G3BP1-depleted group than control group. Scale bar, 30 μm. H&E staining of xenografts was used to assess the tumor sizes of the two groups. The areas marked by blue circles were the xenograft in mice tongues. T, Tumor. Scale bar, 100 μm. (**H**) The statistical results of IHC and H&E staining images were shown via the bar graphs. (*, *p* < 0.05 and **, *p* < 0.01).

In this part, we verified the pro-tumor effects of SG formation in M2 TAMs in vivo. We established mice xenograft model with lingual implantation of SAS cells (transfected with the luciferase genes) mixed with MDMs-M2 transfected with control or G3BP1 shRNA in mice (Figure 6A) and monitored tumors metastasis to the cervical lymph nodes and SG formation in vivo. After mice were sacrificed, we harvested tongue xenografts and cervical lymph nodes. Compared with those in control mice, tumors in mice implanted with SAS cells mixed with MDMs-M2 expressing G3BP1 shRNA exhibited limited growth and metastatic ability (Figure 6B,C). The average weight and volume of the xenograft tumors and cervical lymph nodes in shG3 group mice were lower than those in control group (Figure 6D,E). In addition, the mice in the shG3 group showed significantly longer survival times (Figure 6F). The immunohistochemical data showed lower expression of G3BP1, CCL13 and CD206 in the group of mice implanted with SAS cells mixed with G3BP1-knockdown MDMs-M2 than in the control group, and smaller tumor sizes were observed in G3BP1-depleted group as revealed by H&E staining (Figure 6G,H). These results demonstrated that SG formation in M2 TAMs elevated the production of CCL13 to promote tumor metastasis in vivo.

### 3.7. High Expression of CCL13 Predicts Shorter Overall Survival of OSCC Patients

**Figure 7:**
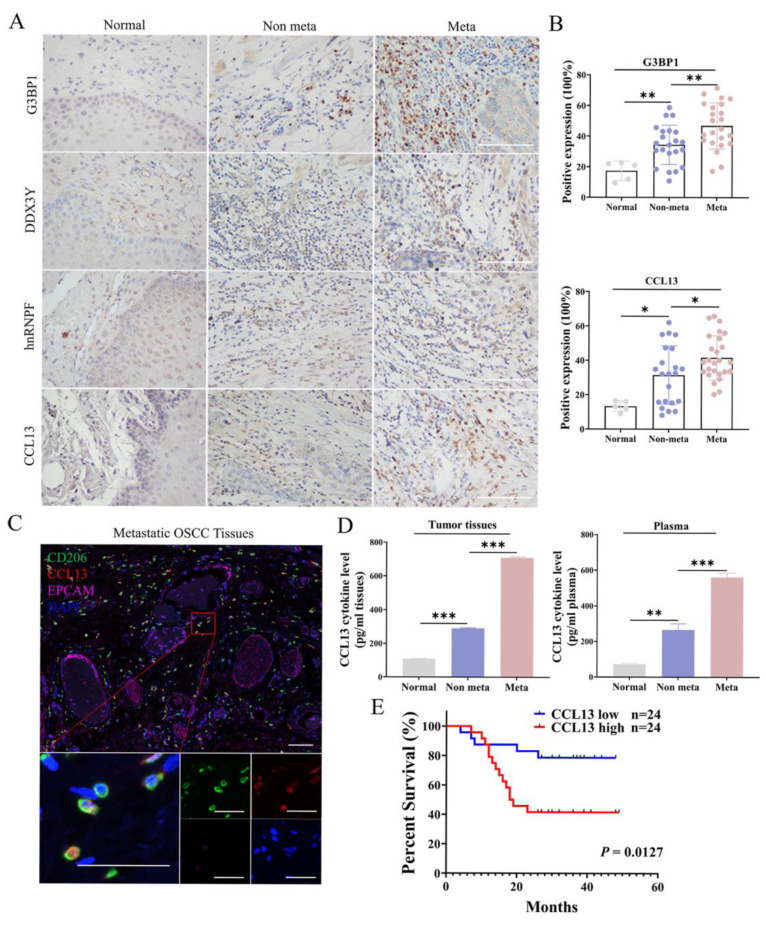
CCL13 was a significant indicator for prognosis of OSCC patients. (**A**,**B**) Representative images of IHC staining showed that metastasis OSCC with higher G3BP1 and CCL13 levels. Scale bar, 10 μm. Statistical analysis of positive staining of indicated proteins in 22 OSCC patients without metastasis or 26 OSCC patients with metastasis. (**C**) The images of mIHC staining showed the co-localization of CD206 and CCL13 in metastatic OSCC tissues. Scale bar, 50 μm. Enlarged pictures were inserted on the bottom. Scale bar, 10 μm. (**D**) CCL13 levels from tumor tissues or serum of OSCC patients were tested by ELISA. Bars correspond to mean ± SD of 3 independent experiments. (**E**) Survival curves of 48 OSCC patients with low or high CCL13 proteins expression. The *p* value was determined by log-rank test. (*, *p* < 0.05; **, *p* < 0.01 and ***, *p* < 0.001).

To correlate the above findings with the physiopathology observed in the clinic, we measured the expression of G3BP1, DDX3Y, hnRNPF and CCL13 in the OSCC tissues. Staining of G3BP1 and CCL13 in metastatic OSCC tissues were stronger than those in OSCC tissues without metastasis (Figure 7A,B). Moreover, CCL13 co-localized with CD206 in metastatic OSCC tissues (Figure 7C). Furthermore, the levels of CCL13 in the serum and tumor tissues of OSCC patients with lymph node metastasis were significantly higher than those in OSCC patients without metastasis (Figure 7D). Finally, a higher level of CCL13 could predict poor survival outcomes of OSCC patients, implying CCL13 can be termed as a potential prognostic indicator for OSCC (Figure 7E). The correlations between CCL13 expression and the clinicopathological features of 48 OSCC patients have been listed in Table S2, which uncovered a positive association between CCL13 expression and lymph nodes metastasis of OSCC (*n* value).

## 4. Discussion

Macrophage-targeted cancer therapy has been proposed as a promising method for tumor elimination [47,48]. A recent study noted that the CD163^+^CD206^−^ TAMs were associated with up-regulated immune signaling and improved survival in gastric cancer, whereas the infiltration of CD68^+^ only and CD163^−^CD206^+^ only TAMs was correlated with a high expression of PD-L1 and tumor immune escape [12]. Both CD163^+^ TAMs and CD206^+^TAMs were correlated with OSCC progression [25,49,50,51]. In this study, we observed that CD206^+^ TAMs promote OSCC metastasis and were linked to poor prognosis of OSCC patients. The main goals of this study were to disclose the novel regulatory mechanisms that M2 TAMs improve OSCC metastasis.

As reported, endoplasmic reticulum stress (ERS) and ISR promoted the expression of stress-associated proteins to support cell survival and cancer progression [32]. Indeed, stress-associated proteins (including G3BP1) in MDMs-M2 were upregulated in our study. G3BP1, one of SG nucleating proteins, is one of “switches” to trigger SG formation, whose inhibition can block SG assembly [38,39]. SG was also deemed as one of membraneless organelles whose formation depends on liquid–liquid phase separation [52]. Researchers found that the SG-associated protein YB-1 mediated SG assembly by binding to the 5′ UTR of G3BP1 mRNA, thus promoting sarcoma invasion and metastasis [29]. In stressed cells, global translation level was limited to prevent the translation of energy-consuming proteins, which facilitated the accumulation of misfolded proteins [43]. As reported, SG formation can be probed in activated T cells in vitro, which has regulated the mRNA translation of the immune checkpoint PD-1 in a microtube-dependent manner [53]. TAMs were also exposed to diverse stressors in TME [27]. In this study, we found SG formed in activated MDMs-M2 in vitro. Interestingly, SG can be detected in MDMs-M2 consecutively treated by inflammatory cytokines cocktails of IL-4/IL-10/IL13, whereas the number of SG induced by positive-controlled sodium arsenate were larger. These results may indicate chronical inflammatory cytokines can stimulate TAMs to induce SG formation. It seems that the acute and strong stress would lead to the incidence of granules larger in size and number inside cells. In order to observe the effects of MDMs-M2 on OSCC cells migration, we established a co-cultured model between MDMs-M2 and OSCC cells utilizing the transwell devices. According to previous studies, SAS cells were acknowledged as a potent OSCC cells with greater metastatic potentials [54], whereas CAL27 cells appeared to be weaker in respect of metastatic potentials. If the pro-metastasis effects of CCL13 can be demonstrated in the two cell lines with distinct metastatic potentials, the hypothesis about pro-tumor properties of CCL13 would be more persuasive. For the next step, we confirmed the roles of SG in improving OSCC metastasis. Inhibition of SG formation attenuated the premetastatic effects of MDMs-M2 according to data in vitro and in vivo and significantly decreased the expression of CD206, which highlighted the critical effects of SG formation.

Based on the above analysis, we motivated to identify the mechanisms by which SG formation enhanced the protumor effects of MDMs-M2. We analyzed the genome-wide changes in G3BP1-knockdown MDMs-M2 and observed that the cluster of differentially expressed genes were related to the inflammatory responses. CCL13 was the most significantly down-regulated cytokine upon G3BP1 knockdown. Chemokines can induce chemotaxis of various cells in the immune system for the recruitment of non-malignant cells into TME and they were also responsible for the migration, invasion, and metastasis of cancer cells [55]. The up-regulation of CCL13 (a member of the CCL chemokine family) was also associated with the development of rheumatoid arthritis and [56] and cancer progression [57]. A prognostic risk model has identified CCL13 as a risk factor for poor prognosis of HNSCC patients [58]. In our study, we confirmed CCL13 promoted OSCC metastasis in vitro and in vivo. The OSCC cells migration were enhanced by approximately two folds by recombinant CCL13 proteins. Collectively, these observations indicated that SG could regulate OSCC metastasis by mediating the production of CCL13.

It was revealed that most differentially expressed genes upon G3BP1 knockdown were associated with the biological processes of protein binding. As one of most vital nucleating proteins, G3BP1 was in charge of searching and sequestering available RNA binding proteins or translationally inhibited mRNA into the shields or cores of SG [40]. Under this circumstance, we identified a series of proteins interacting with endogenous G3BP1 in MDMs-M2 via coimmunoprecipitation and mass spectrometry. We subsequently revealed SG facilitated the connection between G3BP1 and DDX3Y/hnRNPF by coimmunoprecipitation. Disrupting their interaction by knocking down the expression of DDX3Y and hnRNPF decreased the expression of CCL13 and CD206 in MDMs-M2. Moreover, we confirmed that both DDX3Y and hnRNPF were co-localized in SG, but inhibition of SG formation in MDMs-M2 impaired their co-localization. SG formation appeared to reprogram mRNA translation to improve the adaptive response under adverse conditions. SG-associated translation was not rare, whose translation cycles can occur inside SG [44]. SG has been reported to maintain mRNA stability, but the detailed molecular mechanisms remain unclear [59]. In this study, we observed that CCL13 mRNA localized in G3BP1^+^ SG. We wondered whether SG regulates mRNA stability to mediate the CCL13 expression.

Heterogeneous nuclear ribonucleoprotein F (hnRNPF) belongs to the subfamily of ubiquitously expressed heterogeneous nuclear ribonucleoproteins (hnRNPs). The hnRNPs protein family has been reported to promote cancer development [45,60,61]. In our study, we found that knockdown of hnRNPF abolished the migration-promoting abilities of MDMs-M2. Several studies have discovered that hnRNPF regulated gene expression by mediating pre-mRNA alternative splicing via the three repeated quasi-RNA recognition motifs of hnRNPF [62]. The management of mRNA stability is a crucial aspect of gene expression, especially for unstable mRNA transcripts. hnRNPF has been observed to mediate the preservation of targeted mRNAs to decelerate their degradation [45]. In this study, we demonstrated that hnRNPF knockdown reduced the mRNA stability of CCL13 and CD206 in MDMs-M2. SG formation enhanced DDX3Y/hnRNPF-mediated mRNA stability via binding with these transcripts, slowing down their decay to improve their expression. Collectively, SG elevated CCL13 mRNA stability, which was initially sustained by hnRNPF, and therefore upregulated CCL13 expression and promoted OSCC metastasis.

In summary, this study firstly reported that SG formation regulated the expression of CCL13 in M2 TAMs to promote OSCC metastasis. For mechanisms, this study offered new insights into mRNA stabilization regulated by SG. Furthermore, this study provided a novel perspective on TAM-mediated OSCC metastasis and proposed potential targets for TAM-directed cancer therapy.

## 5. Conclusions

This study demonstrated the pro-tumor roles of CCL13 in oral cancer and illustrated that stress granules appeared to a positive regulator of CCL13 expression in M2 TAMs via improving the DDX3Y/hnRNPF-mediated mRNA stability of CCL13. Nonetheless, further studies are still required to disclose more specific molecular mechanism of stress-granule-regulating cancer progression.

## Data Availability

The data that support the findings of this study are available from the corresponding author upon reasonable request.

## Supplementary Materials

All the whole western blot figures can be found in the supplementary materials. The following supporting information can be downloaded at: https://www.mdpi.com/article/10.3390/cancers14205081/s1, Figure S1: The gating strategies of flow cytometry detection of CD163 and CD206 as the surface marker of MDMs-M2; Figure S2: G3BP1 Knockdown inhibited SG formation and CD206 expression; Figure S3: The Venn graph identified a total of 901 differentially expressed genes upon G3BP1 knockdown in MDMs-M2; Figure S4: The candidate proteins interacted with G3BP1 in MDMs-M2; Figure S5: Knockdown of hnRNPF decreased the mRNA stability of CCL13 and CD206. Table S1: Candidate proteins connecting with endogenous G3BP1; Table S2: Correlations Between Clinicopathologic Characteristics of OSCC Patients and the Protein Expression of CCL13; Table S3: Sequences of siRNAs, RT-qPCR primers and Plasmid constructs used in experiments. The supplemental materials and methods, as well as supplemental figure legends were also included in this link URL [63].

## Abbreviations

SG: Stress Granule; ISR: integrated stress response; TME: Tumor microenvironment; GO analysis: Gene Ontology analysis; TAM: Tumor-associated macrophage; MDM: Monocyte derived macrophage; OSCC: Oral Squamous Cell Carcinoma; DDX3Y: DEAD-box helicase 3 Y-linked; G3BP1: G3BP stress granule assembly factor 1; hnRNPF: Heterogeneous nuclear ribonucleoprotein F; rhCCL13: recombinant human CCL13 proteins; SAS-Luci: SAS cell lines expressing luciferase gene.
