# Supplementary material for: Tumor-Associated Macrophages Promote Metastasis of Oral Squamous Cell Carcinoma via CCL13 Regulated by Stress Granule
Source: Cancers (Basel). 2022 Oct 17;14(20):5081. doi: 10.3390/cancers14205081 (PMC9657876; doi:10.3390/cancers14205081)
Supplement: Supplementary file 1 [file cancers-14-05081-s001.zip › cancers-1931011 supplyment figures.pdf]

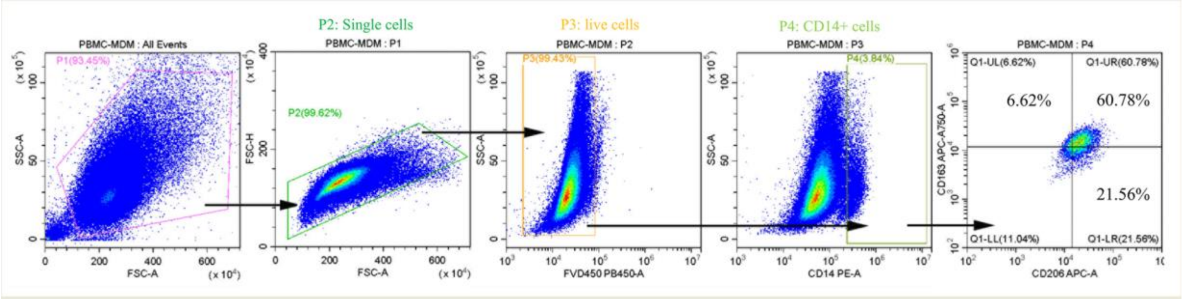

Figure S1. The gating strategies of flow cytometry detection of CD163 and CD206 as the surface marker of MDMs-M2.

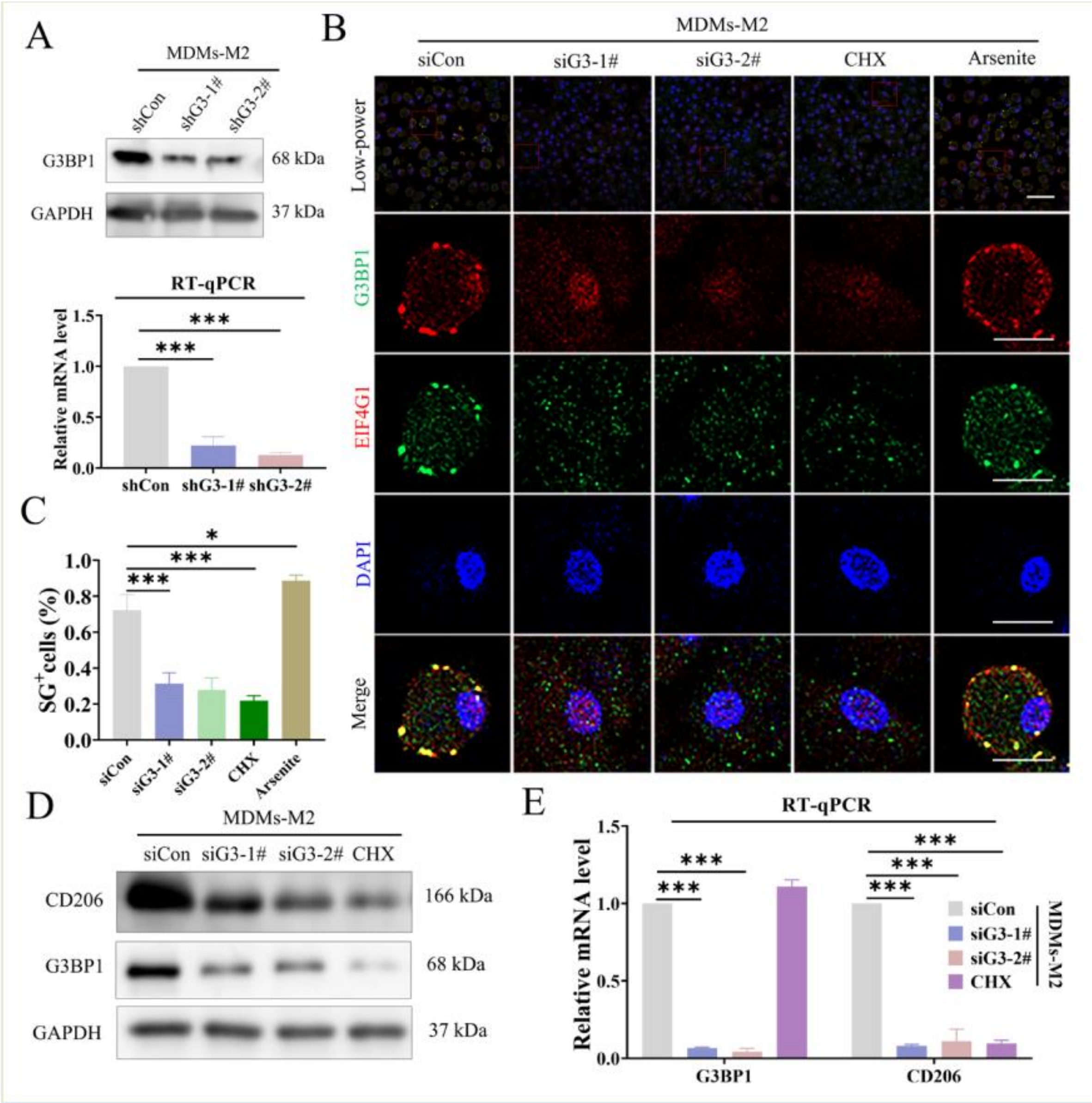

Figure S2. G3BP1 Knockdown inhibited SG formation and CD206 expression. **(A)** The effects of G3BP1 knockdown through stable G3BP1 shRNA transfection were testified by Western blot and RT-qPCR. **(B,C)** Immunostaining of G3BP1

and EIF4G1 in indicated macrophages in vitro verified the effects of inhibiting SG formation via knockdown of G3BP1. Scale bar: 50 um. **(D,E)** Protein expression and mRNA level of CD206 after G3BP1 knockdown in MDMs-M2 via Western blot analysis and RT-qPCR. All experiments were performed in triplicate, and the data are presented as the means ± SD. (*, p<0.05; **, p<0.01 and ***, p<0.001).

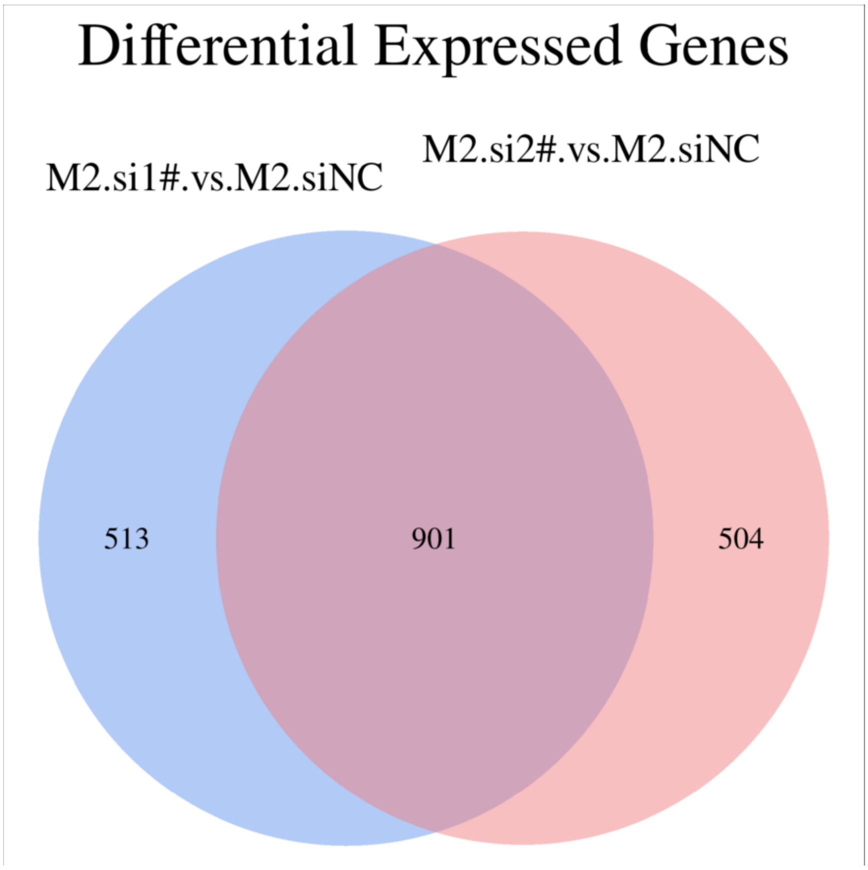

Figure S3. The Venn graph identified a total of 901 differentially expressed genes upon G3BP1 knockdown in MDMs-M2.

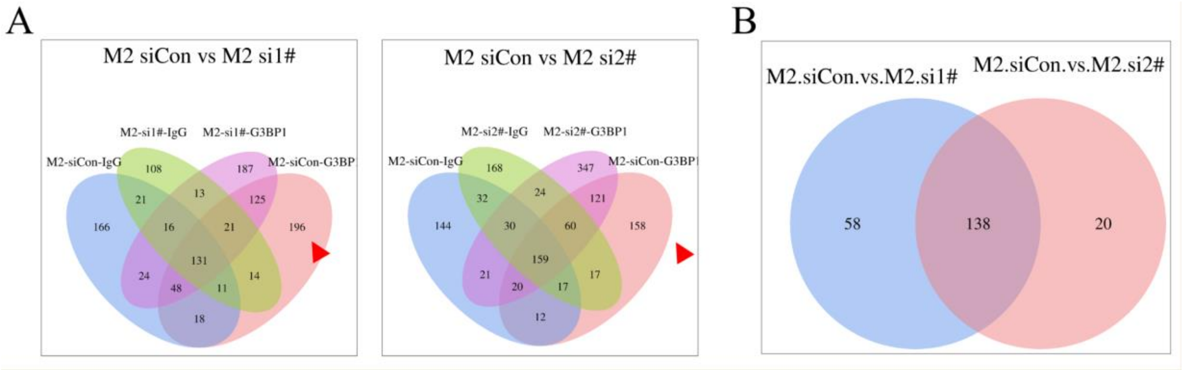

Figure S4. The candidate proteins interacted with G3BP1 in MDMs-M2. **(A)** The lysates from MDMs-M2 transfected with control, or G3BP1 siRNA were performed to coimmunoprecipitation using rabbit isotype IgG or G3BP1 antibody. Then mass spectrometry were used to detect the proteins connecting with G3BP1. Venn graph identified differential proteins upon control MDMs-M2. **(B)** Venn graph identified a total of 138 differential proteins upon controlled MDMs-M2.

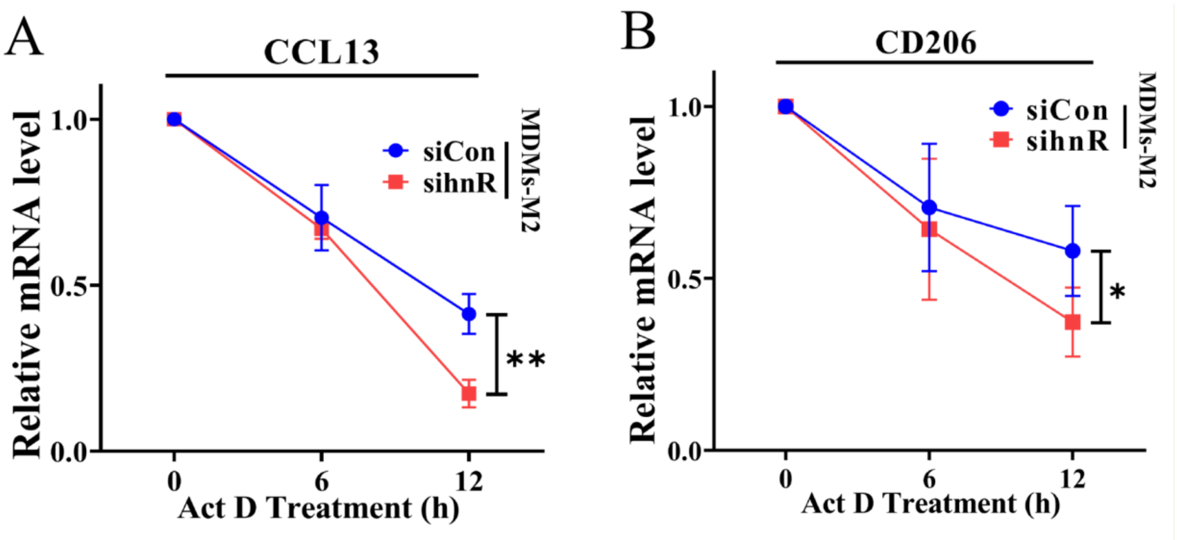

Figure S5. Knockdown of hnRNPF decreased the mRNA stability of CCL13 and CD206. **(A,B)** MDMs-M2 were transiently transfected with control or hnRNPF siRNA and then treated with Actinomycin D for 0h, 6h, 12h. The CCL13 and CD206 mRNA level were determined using RT-qPCR. The GAPDH mRNA level was used as a loading control. The values represented the mean ± SD of 3 independent experiments. (*, $p<0.05$ and **, $p<0.01$).
