# Supplementary material for: Tumor-Associated Macrophages Promote Metastasis of Oral Squamous Cell Carcinoma via CCL13 Regulated by Stress Granule
Source: Cancers (Basel). 2022 Oct 17;14(20):5081. doi: 10.3390/cancers14205081 (PMC9657876; doi:10.3390/cancers14205081)
Supplement: Supplementary file 1 [file cancers-14-05081-s001.zip › Supplementary materials and methods.pdf]

Supplementary materials and methods

Cell culture

The human oral squamous cell carcinoma cell lines (CAL27 and SAS) were obtained from the American Type Culture Collection (ATCC). None of these cell lines were contaminated with mycoplasma. CAL27 and SAS cells were cultured as previously described [1]. The SAS were transfected with lentivirus vector with U6/Luciferase17&Puro (Gene Pharma, #T1722) containing control shRNA and selected with puromycin to gain SAS cell lines expressing luciferase gene (SAS-Luci).

Antibodies

Anti-CD206 antibodies were purchased from Cell Signaling Technology (#91992S, 1:1,000 for IHC, mIHC, IF, or 1: 5,000 for Western blot analysis); anti-G3BP from Abcam, (ab181150, 1:1,000 for IHC, mIHC and IF, or 1:5,000 for Western blot analysis, 1:50 for IHC); anti-DDX3Y from Proteintech (14041-1-AP, 1:400 for IHC and IF, or 1:4,000 for Western blot analysis), anti-hnRNPUL1 from Proteintech (10578-1-AP, 1:4,000 for Western blot analysis), and anti-hnRNPF from Genetex (GTX114476, 1:500 for IHC, mIHC and immunofluorescence, or 1: 3,000 for Western blot analysis). Anti-CCL13 (ab224593 and ab92368) antibodies were purchased from Abcam (1:500 for IHC, mIHC and immunofluorescence, or 1:5,000 for Western blot analysis). Anti-EPCAM was purchased from Abcam (ab213500, 1:1,000 for mIHC).

Immunohistochemistry (IHC) and tissue immunofluorescence staining (IF)

The dissected human or mouse primary tumor tissues were prepared for immunohistochemistry and immunofluorescence analysis as described in other study

[2, 3]. Briefly, after incubating with primary antibodies, the sections were detected with GTVisionTM Detection System (Gene Tech, GK500710) according to the manufacturer's instructions. For the tissue immunofluorescence analysis, sections were incubated with secondary antibody of Alexa Fluor 594 goat anti-mouse IgG (ZS, ZF-0513) or Alexa Fluor 488 goat anti-rabbit IgG (ZS, ZF-0511) posterior to primary antibody incubation.

The statistical analysis of IHC staining were performed with Image J software (1.52k, USA) by calculating the comprehensive percentage depended on cell density and intensity with positive expressions per 20× field.

Cell Immunofluorescence (IF)

The cell IF of macrophages were performed as showed in other study[4]. The slides were examined using a fluorescence microscope (Zeiss LSM 880 with airyscan). The number of SG-bearing cells was quantified using the image J software. IF images were randomly analyzed with a 20× objective in 3 different fields per image. The cells obtaining at least two granules will be deemed as SG positive cells.

Multiplex IHC imaging and inForm analysis

The mIHC Slides were imaged using a Vectra microscope. Whole slide scans were performed using the 10× objective lens. Regions of interest (ROIs) were selected with fixed-size stamps in Phenochart (PerkinElmer), based on the previously acquired whole slide scan images. The viable regions in each specimen were selected with minimal overlap. Selected images were analyzed with inForm for tissue-component segmentation of tumor-cell ($EPCAM^{+}$) and stroma ($EPCAM^{-}$) regions and cell

phenotyping.

Macrophages culture

Peripheral blood monocytes (PBMC) from healthy donors were isolated by Ficoll density gradient centrifugation (GE hyclone, 17-1440-03-1). Monocytes were obtained through PBMC using human CD14 Microbeads (Miltenyi Biotec, 130-050-201) according to the manufacturer's instructions. Monocytes were subsequently cultured for 7-10 days in medium (RPMI medium 1640, GIBCO) with 10% FBS (Biological Industries, 04-001-1A) and 50 ng/ml recombinant human M-CSF (PeproTech, AF-300-25-100) to generate Monocyte-Derived Macrophages (MDMs). M1 or M2-like macrophages were polarized using MDMs via incubating with 40 ng/ml recombinant human IL-4 (PeproTech, 200-04-500) and IL-13 (PeproTech, 400-16-100) or Lipopolysaccharide (LPS) and IFN-γ (PeproTech, AF-300-02B-100). No presence of Mycoplasma was found according to Mycoplasma Detection Kit (Southern Biotech, 13100-01).

Flow Cytometry detection of Macrophages

Macrophages polarized by rh IL-4 and rh IL-13 in vitro were resuspended in flow cytometry buffer containing with 2% FBS and 50 mM EDTA at a concentration of 1 million cells per ml and stained with Ghost Dye™ Violet 450 (1: 500, Tonbo Biosciences, 13-0863-T100) for 10 min and rinsed for three times using flow cytometry buffer. Next the cells were blocked with Fc Receptor Blocking Solution (Trustain FcX, BioLegend, 422301) for 10 min on ice before staining with antibody panels. Samples were stained with primary antibody for 30 min on ice: CD14 (PE anti-human CD14,

301805), CD163 (APC/Cy7 anti-human CD163, 333622), CD206 (APC anti-human CD206, 321109) and subsequently washed twice with flow cytometry buffer. Gating for macrophage markers was performed using fluorescence minus one (FMO) control. Flow cytometry was performed on a FACS Canto II (BD Biosciences). All flow cytometry data reported in this work were analyzed using Flow jo.The gating strategies included: ①Ghost Dye$^-$, CD14$^+$ and CD163$^+$CD206$^-$; ②Ghost Dye$^-$, CD14$^+$, and CD163$^+$CD206$^+$; ③Ghost Dye$^-$, CD14$^+$ and CD163$^-$CD206$^+$.

Preparation of Conditioned Medium (CM) from macrophages

Macrophages were cultured in 250 mL flasks in regular RPMI 1640 medium at 80% confluence. 10 mL RPMI 1640 supplemented with 10% FBS was added to each flask and recollected 48 hours later to obtain macrophage-primed conditioned medium. CM was obtained by mixing the macrophage-primed medium with regular medium (v/v = 1:1). These macrophage CM were used to treat the OSCC cell lines in vitro.

Fluorescence in situ hybridization

Cells were fixed with 4% paraformaldehyde for 15 min then permeabilized with -20 °C methanol for 5 min. Cells were incubated overnight in 70% ethanol at 4 °C. The following day, cells were washed twice with 2× saline-sodium citrate (SSC, Ambion, Waltham, MA USA), blocked in hybridization buffer (Sigma, Saint Louis, MO, USA) for 30 min. Hybridization was performed using 2 ng/μl of MCP-4 probe conjugated with Cy5 fluorescein diluted in hybridization buffer at 37°C, then followed by immunostaining as described above.

siRNA transfection for macrophages

The specific siRNAs targeting G3BP1, DDX3Y and hnRNPF were obtained from GenePharma Biotec (Suzhou, China). siRNA transfection was performed using Lipofectamine RNAiMAX (Invitrogen, USA, 13778150) according to the manufacturer's protocol. The targeting sequences were listed in the supplementary Table S2.

Plasmid construct

To generate and G3BP1 knockdown stable clones, 293 T cells were transfected with lentiviral vectors pGPU6/GFP/Neo with G3BP1 or Control shRNA, together with lentivirus packaging plasmids (psAX2 and pMD2G) for 48 h using Lipofectamine 3000 and p300 (Invitrogen, L3000015). pMD2.G and psPAX2 were used as packaging vectors which were gifted by Professor Chen Weiliang (Sun Yat-sen Memorial Hospital, Sun Yat-sen University, China). Human G3BP1 short hairpin RNAs (shRNAs) were also purchased from GenePharma biotec. The lentivirus supernatant was collected and then added to culture medium of MDMs-M2 for shRNA transduction. Two days after infection with lentivirus supernatant, stable clones were selected with 100 μg/ml Geneticin (Procell, PB180125, Wuhan) for 10 days and puromycin-resistant cells were collected as G3BP1-depleted MDMs-M2. All the constructs were confirmed by both DNA sequencing and diagnostic digestion. The sequences of the shRNA were listed at supplementary Table S2.

RNA sequencing

RNA was extracted using TRIzol (Thermo Fisher Scientific). After removing rRNA, RNAs were fragmented, and reverse transcribed using random primers. cDNAs

were ligated with adaptors, amplified via PCR, and then sequenced with an Illumina sequence analyser. Filtered sequences were aligned with HISAT, and gene expression was analyzed with DEGseq software. Differentially expressed genes were subjected to GO term and Kyoto Encylopedia of Genes and Genomes (KEGG) pathway analysis. Bioinformatic analysis was performed using the OmicStudio tools available at https://www.omicstudio.cn/tool.

Coimmunoprecipitation and mass spectrometry

For immunoprecipitation, the protein supernatants from MDMs-M2 transfected with control or G3BP1 siRNAs were first incubated with Pierce™ Protein A/G Magnetic Beads (Invitrogen, 88802) overnight at 4 °C, and the precipitates were washed three times with wash buffer (Tris-buffered saline (TBS; 28360) containing 0.05% Tween 20 detergent). To detect endogenous interactions, the clarified supernatants were incubated with antibodies against G3BP1 at room temperature for 2 h. After washing, the samples were digested, and the peptides were analyzed by mass spectrometry or western blotting. Mass spectrometry analysis was performed by the Bioinformatics and Omics Center, Sun Yat-Sen Memorial Hospital, Sun Yat-Sen University. Finally, the “Wu Kong” platform (https://www.omicsolution.com/wkomics/main/) was used for relevant GO or KEGG analysis with the mass spectrometry data.

Transwell migration assay

The Transwell invasion assays were performed as previous described [5, 6]. The CCL13 antibody (GeneTex, GTX52589) were used to block their effects on OSCC cells

migration. For the co-culture model between OSCC cells and indicated macrophages, macrophages were placed into lower chambers added by same complete medium with upper chamber and cultured in a 37 °C incubator for 24 hours. The samples were observed under a microscope and imaging and quantification were performed using image J software.

The co-culture model between M2 macrophages and CAL27/SAS cells using transwell devices were consisted of 0.8μm-chambers (upper chambers) and the 24-well cell culture plates (lower chambers). The M2 macrophages were inoculated and cultured in lower chambers 24 hours before the co-culture experiment. On the onset of formal experiment, the CAL27/SAS cells were inoculated in upper chambers. Then the upper chambers covered with CAL27/SAS cells would be placed into the lower chambers with macrophages (avoiding the formation of air bubble at the interface between upper and lower chambers) and continued to culture 24 hour. This was actually a chemotaxis experiment. The chemokines or cytokines produced by M2 macrophages in lower chambers can attract the mobility of CAL27/SAS cells into the lower chambers, thus demonstrating the pro-migration effects of M2 macrophages. In this study, knockdown of G3BP1 inhibited the secretion of CCL13 from M2 macrophages, and therefore blocked the migration of CAL27/SAS cells into the lower chambers.

Wound healing assay

This experiment was performed as previously described [6]. Images were taken at 0 and 24 hours after wounding scratch. The healing percent of wounds can be calculated via the formulas: Healing percent = (wound areas in at 0 hour − wound areas in at 24

hour) / wound areas in at 0 hour × 100%.

ELISA

Serums of OSCC patients were obtained from peripheral blood by centrifuge at 4°C, 2000g, 10 min. Primary OSCC tissues were cut into small pieces with 4 mm diameter and cultured in incubator at 4°C overnight. The culture supernatants of tumor tissues were collected and centrifuged for ELISA test. Macrophages were generated as described above and subsequently transfected with G3BP1 or control siRNA for 48 hours. Supernatant from indicated macrophages was collected to assess the production of CCL13 using Human MCP-4/CCL13 ELISA Kit (Elabscience, E-EL-H1159c) according to manufacturer's instructions.

H&E staining

H&E staining was performed using Hematoxylin-Eosin staining Kit (Meilunbio, MB9898) according to manufacturer's instruction. The area of the tumor lesions was calculated using image J software. The workflow for area measurement of metastatic tumors in mice neck requires two steps: 1. Select the region of interest (ROI) and perform image segmentation; 2. Measure the area of positive staining parts. Use tools such as rectangle, circle or other ways to select the parts of images wanted by us, and then Measure their areas according to the tips of software. A formula was used to compared the percent of xenografts area to the area of whole tongue tissues in different group: (areas of xenografts / areas of tongue)×100%.

Orthotopic metastasis model in the mouse tongue

All animal experiments were approved by the Institutional Animal Care and Use

Committee in our hospital. SAS-Luci cells (2.0×10^6) alone and SAS-Luci cells (2.0×10^6) mixed with stable G3BP1-knockdown MDMs-M2 (1.0×10^6) or control MDMs-M2 (1.0×10^6) were injected into the tongues of BALB/c nude mice (female, 4 weeks old) to establish tumor xenografts (n=6). When the xenografts were palpable (approximately 0.5 cm in diameter), intratumor injection of the indicated cytokines was performed as described in another study [2]. In some experiments, mice were intraperitoneally injected with 15 mg/ml D-Luciferin and potassium salt (Aladdin, D266332) and were then observed on 18 days after tumor transplantation with a Spectrum in vivo imaging system (IVIS Spectrum, PerkinElmer, USA). Bioluminescence of the tongues and cervical lymph nodes were quantified as total flux (photons/s). Living Image software 4.4 (Caliper Life Sciences) was used to determine the integrated flux of photons (photons/s) within each region of interest. Tumor volumes were monitored as described previously [2]. Animals were sacrificed when the xenografts reached 0.8 cm in diameter. Tumor xenografts and cervical lymph nodes of the mice were harvested for further evaluation. Paraffin sections of harvested tissues were used for H&E staining or immunohistochemical analysis.

Reference

1 Zhang S, Ma H, Zhang D, Xie S, Wang W, Li Q *et al*. LncRNA KCNQ1OT1 regulates proliferation and cisplatin resistance in tongue cancer via miR-211-5p medi ated Ezrin/Fak/Src signaling. *Cell Death Dis* 2018; 9: 742.

2 Chen J, Yao Y, Gong C, Yu F, Su S, Chen J *et al*. CCL18 from tumor-associated macrophages promotes breast cancer metastasis via PITPNM3. *Cancer Cell* 2011; 19: 541-555.

3 Chen F, Chen J, Yang L, Liu J, Zhang X, Zhang Y *et al*. Extracellular vesicle-packaged HIF-1¶¡-stabilizing lncRNA from tumour-associated macrophages regulates aerobic glycolysis of breast cancer cells. *Nat Cell Biol* 2019; 21: 498-510.

4 Yang P, Mathieu C, Kolaitis RM, Zhang P, Messing J, Yurtsever U *et al*. G3BP1 Is a Tunable Switch that Triggers Phase Separation to Assemble Stress Granules. *Cell* 2020; 181: 325-345.e328.

5 Zhou B, Zhuang XM, Wang YY, Lin ZY, Zhang DM, Fan S *et al*. Tumor necrosis factor ¶¡ induces myofibroblast differentiation in human tongue cancer and promotes inv asiveness and angiogenesis via secretion of stromal cell-derived factor-1. *Oral Oncol* 2015; 51: 1095-1102.

6 Wang Y, Lin Z, Sun L, Fan S, Huang Z, Zhang D *et al*. Akt/Ezrin Tyr353/NF-¶ B pathway regulates EGF-induced EMT and metastasis in tongue squamous cell carci noma. *Br J Cancer* 2014; 110: 695-705.
