# Supplementary material for: Tumor-Associated Macrophages Promote Metastasis of Oral Squamous Cell Carcinoma via CCL13 Regulated by Stress Granule
Source: Cancers (Basel). 2022 Oct 17;14(20):5081. doi: 10.3390/cancers14205081 (PMC9657876; doi:10.3390/cancers14205081)
Supplement: Supplementary file 1 [file cancers-14-05081-s001.zip › Supplementary tables.pdf]

Table S1. Candidate proteins connecting with endogenous G3BP1.

Gene Symbol	Biological Process	Cellular Component	Molecular Function
BSQV gp1; flavivirus polyprotein gene	cell organization and biogenesis; metabolic process; regulation of biological process; **response to stimulus**; transport	extracellular; membrane	catalytic activity; metal ion binding; nucleotide binding; protein binding; RNA binding; structural molecule activity
C1QBP	cell death; cell organization and biogenesis; defense response; metabolic process; regulation of biological process; **response to stimulus**	cell surface; cytoplasm;cytosol; extracellular; membrane; mitochondrion; nucleus; organelle lumen	protein binding; RNA binding
CHD2	cell differentiation; cell organization and biogenesis; metabolic process; regulation of biological process; **response to stimulus**	nucleus	catalytic activity; DNA binding; nucleotide binding; protein binding; RNA binding
DDX3Y	metabolic process;regulation of biological process; **response to stimulus**	cytoplasm; cytosol; membrane; nucleus	catalytic activity; DNA binding; nucleotide binding; RNA binding
G3BP2	cell organization and biogenesis; regulation of biological process; **response to stimulus**; transport	cytoplasm; cytosol	protein binding; RNA binding; structural molecule activity
HNRNPF	metabolic process; regulation of biological process; **response to stimulus**	cytosol; membrane; nucleus; spliceosomal complex	protein binding; RNA binding
HNRNPUL1	metabolic process; regulation of biological process; **response to stimulus**	nucleus	protein binding; RNA binding
MKI67	cell proliferation; metabolic process; regulation of biological process; **response to stimulus**	chromosome; cytoplasm; membrane; nucleus	DNA binding; nucleotide binding; protein binding; RNA binding
MSN	cell differentiation; cell organization and biogenesis; cell proliferation; cellular component movement; regulation of biological process; **response to stimulus**	cell surface; cytoplasm; cytoskeleton; cytosol; membrane; nucleus	protein binding; RNA binding; structural molecule activity

Table S2. Correlations Between Clinicopathologic Characteristics of OSCC Patients and the Protein Expression of CCL13

	CCL13 Staining		*P* value
	Low (%) 24	High (%) 24	
Age			
>60	9 (64.29%)	5 (35.71%)	0.204
≤60	15 (44.12%)	19 (55.88%)	
Gender			
Male	18 (60.00%)	12 (40.00%)	0.0736
Female	6 (33.33%)	12 (66.67%)	
Smoking			
Yes	7 (70.00%)	3 (30.00%)	0.1551
No	17 (44.74%)	21 (55.26%)	
T value			
T1, T2	24 (100.00%)	24 (100.00%)	-
T3, T4	0 (0.00)	0 (0.00)	
N value			
N0	16 (66.67%)	8 (33.33%)	0.0209*
N1, N2, N3	8 (33.33%)	16 (66.67%)	
Distant Metastasis			
M0	24 (51.06%)	23 (48.94%)	0.3122
M1	0 (0.00%)	1 (100.00%)	
Stage			
I, II	13 (61.90%)	8 (38.10%)	0.1457
III, IV	11 (40.74%)	16 (59.26%)	
Grade			
Poor	0 (00.00%)	6 (100.00%)	
Moderate	13 (59.09%)	9 (40.91%)	0.0875
Well	11 (55.00%)	9 (45.00%)	

*$P < 0.05$ via using the Chi-square Test.

Table S3. Sequences of siRNAs, RT-qPCR primers and Plasmid constructs used in experiments

	Name		Oligo sequences (5' to 3')
siRNA	G3BP1 siRNA and control siRNA	siRNA-1#	GCAACAGUAUUUCGGUAUAdTdT
		siRNA-2#	UAAUUUCCCACCACUGUUAAUGCGC
		control	UUCUCCGAACGUGUCACGUdTdT
	DDX3Y siRNA and control siRNA	siRNA-1#	GCAAGCAAAGGGCGUUAUA
		siRNA-2#	GGUUAGACUUCUGCAAAUA
		control	GCACAAGCUGGAGUACAACUACATT
	hnRNPF siRNA and control	siRNA-1#	CCGCAGGUGUCCAUUUCAUTT
		siRNA-2#	GGUACAUUGAGGUGUUCAATT

	siRNA	control	GCACAAGCUGGAGUACAACUACATT
qPCR	G3BP1	forward	ACATAGCTCAGACAGTACAGGAA
		reverse	GCACTCTTTGATCCCGCTG
	CD206	forward	TCCGGGTGCTGTTCTCCTA
		reverse	CCAGTCCGTTTTTGATGGCACT
	CCL13	forward	CTCAACGTCCCATCTACTTGC
		reverse	TCTTCAGGGTGTGAGCTTTCC
	CCL22	forward	ATCGCCTACAGACTGCACTC
		reverse	GACGGTAACGGACGTAATCAC
	GAPDH	forward	GGAGCGAGATCCCTCCAAAAT
		reverse	GGCTGTTGTCATACTTCTCATGG
	CCL23	forward	CATCTCCTACACCCCACGAAG
		reverse	GGGTTGGCACAGAAACGTC
	TNFSF8	forward	AGAGGACGGACTCCATTCCC
		reverse	GGTAGGCCCATGACTTCTTGAA
	IL-16	forward	ACGAAGCTACTTGACGAAAAGAC
		reverse	GTTTCAGCAGAACCATTTGCAG
	DDX3Y	forward	GAGGAAACACGGGGATTAACTT
		reverse	GGAGTAGGACGAGTATAGCGAG
	hnRNPF	forward	GAAGGCTCTAGGGAAACACAAG
		reverse	CACGGACATGAACTTCAGAGG
shRNA	G3BP1 shRNA and Control shRNA	shCon	GCUUCUACCAAAUACACUUGA
		shG3BP1-1#	GGAGATTCATGCAAACGTTTG
		shG3BP1-2#	GCCTGTTCAGAAAGTCCTTAG
